# Quality of life among home office-based university administrative personnel

**DOI:** 10.47626/1679-4435-2021-951

**Published:** 2024-08-05

**Authors:** Poliana Simas Magalhães, Mila Alves de Matos Rodrigues, Rizia Rocha Silva, Lucas Lima Galvão, Tharcilla Nascimento da Silva Macena, Claudio Andre Barbosa de Lira, Douglas de Assis Teles Santos

**Affiliations:** 1 Colegiado de Ciências Biológicas, Campus X, Universidade do Estado da Bahia (UNEB), Teixeira de Freitas, BA, Brasil; 2 Laboratório de Avaliação do Movimento Humano, Faculdade de Educação Física e Dança, Universidade Federal de Goiás, Goiânia, GO, Brasil; 3 Departamento de Ciências do Esporte, Universidade Federal do Triângulo Mineiro, Uberaba, MG, Brasil; 4 Colegiado de Educação Física, Campus X, UNEB, Teixeira de Freitas, BA, Brasil

**Keywords:** occupational health, universities, COVID-19, saúde do trabalhador, universidades, COVID-19

## Abstract

**Introduction:**

The integration of remote work into higher education institutions has led to increased administrative activities and has affected quality of life, especially at work.

**Objectives:**

To verify the influence of income on the quality of life and quality of work life of administrative personnel of a higher education institution who worked remotely during the COVID-19 pandemic.

**Methods:**

This descriptive cross-sectional study included 18 employees who worked via home office in the teaching, research, and/or extension sectors of a public university in Bahia, Brazil. Data were collected through an online questionnaires regarding sociodemographic data, quality of life (36-item Short-Form Health Survey), and quality of work life (Total Quality of Work Life).

**Results:**

Older employees had higher income. Higher income was associated with higher quality of life scores for physical, emotional, and mental health, while lower income was associated with better scores for interpersonal relationships and work hours. There was a high prevalence of COVID-19 infection. Quality of life and quality of work life were considered worse than in the pre-pandemic period.

**Conclusions:**

Income influenced several aspects of quality of life and quality of work life (physical, emotional, and mental health, as well as interpersonal relationships and work hours) among university administrative personnel who worked remotely working during the COVID-19 pandemic.

## Abstract (pt)

**Introdução:**

A implementação do trabalho remoto nas instituições de ensino superior tem provocado um aumento nas atividades administrativas e causado diversos efeitos na qualidade de vida, sobretudo em relação ao trabalho.

**Objetivos:**

Verificar a influência da renda na qualidade de vida e na qualidade de vida no trabalho dos servidores técnicos administrativos de uma instituição de ensino superior em *home office* durante a pandemia da covid-19.

**Métodos:**

Tratou-se de um estudo transversal descritivo com 18 servidores técnicos administrativos em *home office*, lotados nos setores de ensino, pesquisa e/ou extensão de uma universidade pública no estado da Bahia, Brasil. Os dados foram coletados por meio de um questionário on-line no qual se investigou dados sociodemográficos, a qualidade de vida (Short-Form Health Survey) e a qualidade de vida no trabalho (Total Quality of Work Life).

**Resultados:**

Os técnicos administrativos mais velhos possuíam maior renda. A maior renda esteve associada a melhores escores na qualidade de vida para os aspectos físicos, emocionais e de saúde mental, enquanto aqueles com menor renda obtiveram melhores escores na qualidade de vida no trabalho para os aspectos relações interpessoais e jornada de trabalho. Houve elevada prevalência de contaminação pela covid-19, e a qualidade de vida e a qualidade de vida no trabalho foram consideradas piores em comparação ao período pré-pandemia.

**Conclusões:**

A renda influencia em aspectos físicos, emocionais e de saúde mental, bem como em aspectos de relações interpessoais e jornada de trabalho, impactando a qualidade de vida e a qualidade de vida no trabalho de técnicos administrativos em *home office* durante a pandemia da covid-19.

## INTRODUCTION

Recognized by the World Health Organization in March 2020, the COVID-19 pandemic has had a great impact on the world population’s health and quality of life (QoL).^1^ Due to the rapid evolution and high transmissibility of the virus, social isolation became the main coping and prevention strategy of government institutions in many countries to slow the spread of the pandemic.^1^

Institutions began providing services via information and communication technologies in a home office model^2^ while face-to-face activities were suspended, which directly affected the daily routines of the entire academic community: professors, students, and administrative staff. In southern regions of Bahia, Brazil, activities were suspended by ordinance (Órgãos Deliberativos da Administração Superior, no.133^3^ and 224^4^).

The Brazilian Consolidated Labor Laws (No. 13,467, July 13, 2017) recognized remote work (“home office”) as a work condition.^5^ Despite being unregulated, participation in remote work is becoming significant. According to the Brazilian Institute of Geography and Statistics’ National Household Survey, 24.7% of public sector employees were working remotely in June 2020.^6^

In higher education institutions, for example, remote work has led to increased administrative activities, which has affected the health of administrative personnel. This was demonstrated in a study conducted during the pandemic on perceived QoL based on job satisfaction, especially socioeconomic aspects, lifestyle habits, and health.^7^

According to Ribeiro & Mancebo,^8^ technological development and increased competitiveness on a global scale have significantly affected economic, social, cultural, and political activities, as well as the job market and the day-to-day affairs of organizations. Educational institutions have not escaped these changes, which have affected the health and QoL of the academic community in general.^9^

According to a 2018 higher education census by the Anísio Teixeira National Institute of Educational Studies and Research, there are 2.537 higher education institutions in Brazil, of which 7.8% (n = 199) are universities; 53.8% of the universities are public (42.8% of which are state universities).^10^ The administrative staff of these institutes provide the necessary support for academic activities to run smoothly.^10^

Thus, it is important to determine the health status and QoL of these workers, observing basic issues, such as food and nutrition, housing and sanitation, work conditions, continuing education, environmental issues, family and individual social support, lifestyle, and health care, which directly influence individual development.^11^

The World Health Organization defines QoL as the individual perception of one’s position in life in the context of the culture and value systems in which one lives and in relation to one’s goals, expectations, standards, and concerns.^11^ QoL is being increasingly used to assess urban living conditions regarding the health, comfort, and material goods of a given population.^11^

Furthermore, excessive workload, low wages, and high individual or collective demand are sources of dissatisfaction among university employees. If, on the one hand, work can be an important source of satisfaction and health, on the other it can also become a source of unhappiness and illness, especially in unsuitable work environments.^12,13^ This is why studies on quality of work life (QWL) are relevant, since research has indicated that most education professionals experience numerous stressors in the work environment, given the difficulties institutions commonly have in meeting the needs individual employees.^12,13^ QWL is related to the physical, environmental, and psychological aspects of the work environment in an intraand interpersonal relationship process based on mutual respect among employees and between employees and the institution.^12,13^

QWL analysis indicates the general health status of workers in a continuous search for improved standards of well-being that considers, in addition to the abovementioned aspects, economic, social, housing, leisure, physical activity, and nutrition.^12,13^

Promoting QWL leads to greater benefits for employees, universities, and society as a whole.^12,13^ Assessing the QWL of administrative personnel at public higher education institutions is important, given that it can provide a scientific basis for the development of QWL programs for emerging situations.^13^

Therefore, it is important to research different aspects of QWL among administrative personnel, such as their socioeconomic profile and health habits, to provide evidence that can raise awareness about work routines, stimulate healthy habits, and support health prevention and promotion initiatives.

The objective of this study was to determine the influence of income on the QoL and QWL of administrative personnel of higher education institutions who worked from home during the COVID-19 pandemic. We hypothesized that those with higher family income would have better QoL and QWL than those with lower income.

## METHODS

### PARTICIPANTS AND STUDY DESIGN

This cross-sectional study was conducted at a state higher education institution in a municipality in the state of Bahia. The institution had 27 administrative personnel, irrespective of employment relationship (permanent or temporary). Of these, 18 agreed to participate in the study; those who were away from work for any reason were excluded.

A Google Forms link was sent to all academic sectors and was subsequently sent to institutional email addresses between May 2021 and June 2021. Participation was voluntary; only those who provided written consent after reading about the procedures, risks, and benefits of participation were included. All procedures were performed in accordance with the Declaration of Helsinki and were approved by the State University of Bahia Human Research Ethics Committee (no. 44088721.0.0000.0057).

### INVESTIGATED VARIABLES

Self-reported data on age, height, weight (to calculate body mass index), work characteristics (employment relationship and work hours), physical activity level,^14^ and sedentary behavior were collected.^14^ The participants were also asked whether they were currently or had previously been infected by COVID-19 and whether they considered their QoL and QWL to be worse, the same, or better than in the period before the pandemic. The participants’ characteristics are shown in Table 1.

**Table 1:** Participant characteristics

Variables	Total (n = 18)		< Income (n = 9)		higher income (n = 9)		P-value
	Mean ± SD	Median (IQR)	Mean ± SD	Median (IQR)	Mean ± SD	Median (IQR)	
Age years)	35.8 ± 11.7	33.00 (230)	28.2 ± 8.8	26.00 (5.0)	43.4 ± 9.0	46.00 (17.0)	0.002
BM (kg)	68.9 ± 16.7	6500 (170)	64.0 ± 8.3	65.00 (6.5)	73.8 ± 21.6	62.00 (38.5)	0.863
Height (m)	1.6 ± 9.4	1.65 (0.1)	1.6 ± 5.3	1.65 (0.6)	1.6 ± 11.5	1.57 (0.2)	0.134
Work hours/week	35.2 ± 8.1	40.00 (10.0)	32.2 ± 9.7	40.00 (20.0)	38.2 ± 4.8	40.00 (5.0)	0222
SB (min/day)	428.9 ± 183.4	411.00 (251.0)	480.8 ± 207	420.00 (473.0)	377.1 ± 151	394.00 (274.0)	0.242
MVPA (min/week)	670.6 ± 803.3	413.00 (591.0)	516.0 ± 535	411.00 (262.0)	826.0 ± 1.014	400.00(1610.0)	0.931

BM = body mass; MVPA = moderate-to-vigorous physical activity; SB = sedentary behavior.

### INCOME

Income (in BRL) was assessed according to selfreport, with the participants divided into 2 groups based on the median value (BRL 2750.00): lower income (n = 9) and higher income (n = 9) groups.

### QOL

The Short-Form Health Survey (SF-36), developed by Ware & Sherbourne^15^ and validated for Brazilian Portuguese by Ciconelli et al.,^16^ was applied to assess QoL. This 36-item multidimensional instrument quantifies health-related QoL in 8 domains (functional capacity, physical aspects, pain, general health status, vitality, social aspects, emotional aspects, and mental health), which are grouped into 2 dimensions (physical and mental). Scores vary from 0 (worst) to 100 (best), except the health report, which is assessed on a scale from 0 to 5, with higher scores indicating better health.^17^ This instrument has good internal consistency, with a Cronbach’s alpha ranging from 0.76 to 0.90 for all subscales.^18^

### QWL

QWL was assessed with the Total Quality of Work Life (TQWL-42) instrument, which was developed and validated by Pedroso et al.^19^ The instrument comprises 42 questions in 5 spheres that involve 4 aspects each: biological/physiological (aspects: physical and mental disposition, work capacity, health and social assistance services, and rest time), psychological/behavioral sphere (aspects: self-esteem , task significance, feedback, and personal and professional development), sociological/ relational sphere (aspects: freedom of expression, interpersonal relationships, autonomy, and leisure time), economic/political sphere (aspects: financial resources, extra benefits, work hours, and job security), and environmental/organizational sphere (aspects: working conditions, growth opportunities, task variety, and task identity). In addition to these spheres, the instrument also includes a self-assessment of QWL.

All TQWL-42 questions are closed, with a response scale ranging from 1 to 5. To analyze the results, the QWL rating scale is suggested, in which a central point (50) characterizes an intermediate level of QWL. Values in the range of 0 to 25 (or 0 to 1.25) are considered “very unsatisfactory”, 25 to 50 (or 1.26 to 2.5) are “unsatisfactory”, 50 to 75 (or 2.6 to 3.75) are “satisfactory”, and 75 to 100 (or 3.76 to 5) are “very satisfactory”. The instrument has a high level of internal consistency and reliability, with a Cronbach’s alpha of 0.85.^19^

### STATISTICAL ANALYSIS

The statistical analysis was performed in IBM SPSS Statistics 25.0. The Shapiro-Wilk test was used to assess for data normality. The *t*-test for independent samples was used for normally distributed data (height, sedentary behavior, TQWL-42 spheres, and SF-36 domains [functional capacity, pain, general health status, and vitality]). The Mann-Whitney U test was used for non-normally distributed data (age, body mass, physical activity, aspects of the TQWL-42, and the other SF-36 domains [limitation by physical aspects, social aspects, emotional aspects, and mental health]). The data are presented as mean, median, SD, and IQR, absolute values (n), and relative frequency (%). Statistical significance was set at 5%.

## RESULTS

The sample consisted of 18 administrative personnel who worked in the following departments: academic coordination (n = 6; 33%), course committees (n = 5; 28%), libraries (n = 2; 11%), information technology (n = 1; 6%), financial coordination (n = 2; 11%), protocols (n = 1; 6%), and sector management (n = 1; 6%). Regarding the employment relationship, 33% (n = 6) are civil servants with permanent positions and 67% (n = 12) were non-permanent: 11% (n = 2) had commissioned positions, 17% (n = 3) were interns, 33% (n = 6) were hired under the Brazilian Consolidated Labor Laws, and 6% (n = 1) were temporary employees through the Special Administrative Legal Regime. The participants’ characteristics are shown in Table 1.

Table 2 shows the QoL comparison between lower and higher income groups. The higher income group had significantly higher scores in the physical, emotional, and mental health domains. The QWL results between the lower and higher income groups are shown in Table 3. There was no significant difference between the spheres. However, regarding QWL aspects, the lower income group had significantly higher scores for interpersonal relationships (classified as “very satisfactory”) and work hours (classified as “satisfactory”).

**Table 2:** Quality of life (36-item Short-Form Health Survey) comparison among administrative personnel of a public university who worked remotely during the COVID-19 pandemic

Variables	Total (n = 18)		lower income (n = 9)		higher income (n = 9)		P-value
	Mean ±SD	Median (IQR)	Mean ± SD	Median (IQR)	Mean ±SD	Median (IQR)	
Functional capacity	76.7 ± 17.4	78(26)	79.4 ± 17.8	75 (38)	73.9 ± 17.6	80 (30)	0.515
Physical aspects	58.3 ± 33.2	50 (75)	41.7 ± 27.9	50(25)	75.0 ± 30.6	75 (50)	0.050
Pain	61.2 ± 22.4	61 (28)	54.0 ± 26.6	51 (48)	68.3 ± 15.8	62(22)	0.183
General health status	63.3 ± 24.5	67 (45)	54.9 ± 26.6	57(45)	71.8 ± 20.2	77(30)	0.148
Vitality	47.2 ± 24.3	40 (34)	40.0 ± 24.5	35 (30)	54.4 ± 23.1	55 (40)	0.216
Social aspects	61.1 ± 34.8	63(63)	50.0 ± 36.4	50 (63)	72.2 ± 31.1	88(50)	0222
Emotional aspects	51.9 ± 46.1	67(100)	25.9 ± 40.1	0(67)	77.8 ± 37.3	100 (50)	0.019
Mental health	57.8 ± 31.3	68(57)	41.3 ± 32.6	36 (64)	74.2 ± 20.3	72(24)	0.024

**Table 3:** Comparison of Total Quality of Work Life scores among administration personnel of a public university who worked remotely during the COVID-19 pandemic

Variables	Total (n= 18)	lower income (n = 9)	higher income (n = 9)	P-value
	Mean ± SD	Mean ± SD	Mean ± SD	
Spheres Biological and physiological	3.0 ± 0.7	3.2 ± 0.9	27 ± 0.5	0.164
Psychological and behavioral	3.7 ± 0.6	3.7 ± 0.7	3.6 ± 0.5	0.742
Sociological and relational	3.4 ± 0.6	3.5 ± 0.5	3.3 ± 0.6	0.398
Economic and political	3.1 ± 0.5	3.3 ± 0.4	2.9 ± 0.6	0.098
Environmental and organizational	3.5 ± 0.4	3.4 ± 0.4	3.4 ± 0.5	0.797
General	3.3 ± 0.5	3.4 ± 0.5	3.2 ± 0.4	0.253
	**Median (IQR)**	**Median (IQR)**	**Median (IQR)**	**P-value**
Aspects Physical and mental disposition	2.5 (1.5)	3.0 (1.5)	2.5 (0.8)	0.063
Work capacity	4.0 (1.5)	4.0 (1.3)	3.6 (1.8)	0.436
Health and social assistance services	3.3 (1.6)	2.0 (1.8)	2.5 (1.8)	1.000
Rest time	3.0 (1.6)	3.0 (1.8)	2.5 (1.8)	0.222
Self-esteem	3.5 (2.0)	4.0 (1.5)	3.0 (1.5)	0.113
Task significance	4.5 (1.1)	4.0 (0.8)	4.5 (1.0)	0.161
Feedback	4.0 (1.6)	4.0 (2.0)	0.5 (1.3)	0.605
Personal and professional development	3.0 (1.6)	3.0 (1.3)	3.0 (2.0)	0.546
Freedom of expression	3.0 (1.0)	3.0 (0.8)	3.0 (1.5)	0.931
Interpersonal relationships	4.0 (1.1)	4.0 (1.0)	3.5 (1.0)	0.040
Autonomy	3.3(10)	3.0 (1.3)	3.5 (0.8)	1.000
Leisure time	3.0 (1.5)	3.0 (1.0)	2.5 (2.0)	0.546
Financial resources	2.8 (1.0)	3.0 (0.5)	2.5 (1.3)	0.340
Extra benefits	2.3(10)	2.5 (1.3)	2.0 (1.3)	0.546
Day shift	3.5 (1.0)	4.0 (1.3)	3.0 (1.3)	0.019
Job security	4.0 (1.0)	4.0 (1.0)	4.0 (0.8)	0.666
Work conditions	3.5 (1.0)	4.0 (1.0)	3.5 (1.3)	0.730
Growth opportunity	2.5 (1.1)	3.0 (1.3)	2.5 (1.8)	0.297
Task variety	3.5 (1.0)	3.3(10)	4.0 (1.0)	0.222
Task identity	4.5 (0.6)	4.5 (0.8)	4.5 (0.8)	0.666
Self-reported quality of work life	3.0 (1.5)	3.0 (1.7)	3.0 (1.0)	0.297

**Figure 1:**
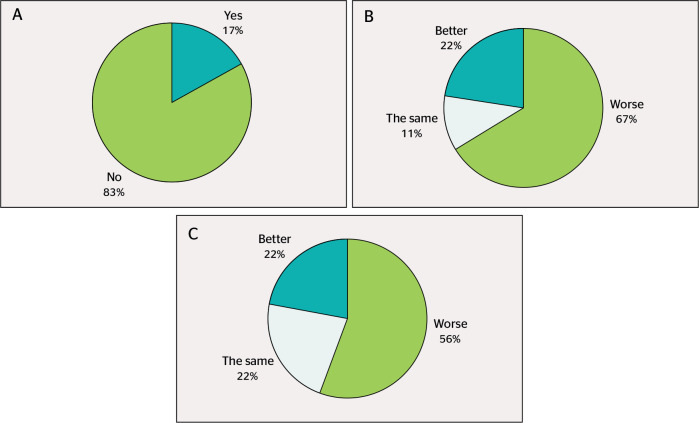
COVID-19 infection prevalence and comparison between quality of life and quality of work life before the pandemic and while working from home. a) Responders who tested positive or not for COVID-19; b) Quality of life before the pandemic; c) Quality of work life compared to the pre-pandemic period.

A total of 17% of the staff had been infected with COVID-19 (n = 3). When asked about their current QoL in comparison to the pre-pandemic period, 22% (n = 4) reported that it was better, 11% (n = 2) the same, and 67% (n = 12) worse. Regarding prepandemic QWL, 22% (n = 4) reported that it was better, 22% (n = 4) the same, and 56% (n = 10) worse (Figure 1).

## DISCUSSION

This study determined the influence of income on QoL and QWL among administrative personnel at a higher education institution who worked from home during the COVID-19 pandemic. The main findings were: i) older personnel had higher income; ii) the higher income group had better QoL scores for the physical, emotional, and mental health domains; iii) the lower income group had better QWL scores for interpersonal relationships and work hours; iv) there was a high prevalence of COVID-19 infection; and v) QoL and QWL were both considered worse than in the pre-pandemic period.

Income allows policymakers to directly identify a country’s economically disadvantaged population.^20^ Our findings showed that older administrative personnel had higher income, which corroborates Brazilian indicators.^21^ Institutionally, the career plan of administrative personnel at the investigated institution involves potential salary increments at specific intervals,^22^ which explains this result.

Other studies have also found a relationship between higher income and higher QoL.^12,13,23^ Pedrolo et al.^13^ analyzed the impact of the COVID-19 pandemic on the QoL of professors at a federal institution, finding a surprisingly good QoL. This is probably due to the fact that they are civil servants with career stability. Mastropietro et al.,^12^ investigated the relationship between income, work, and QoL, finding an association between income and the mental health domain, i.e., those whose income was > 2 times the federal minimum salary were a mean of 4.7% more likely to have the highest mental health scores. Sprangers et al.^23^ found that lower QoL levels are associated with lower income, potentially compromising health conditions and, consequently, QoL.^24^

In addition to high mean values for mental health, Silva & Carvalho^25^ also found similar results for the emotional domain. Santos et al.,^26^ also found a high rate of limitations among professors due to physical and emotional factors. The physical and mental health of civil servants is reflected in professional performance, with inadequate conditions, difficulties in professional relationships, high stress, and workload pressure being risk factors for mental and physical health. In Santa Catarina, Brazil, Serafim et al.^27^ found a relationship between risk factors and work-related illness in state civil servants. It is desirable for employees to enjoy excellent levels of physical and mental health, given that health problems directly affect QoL.

Regarding the finding that QWL was better among administrative personnel with lower income, other studies using the TQWL-42 instrument have found that the interpersonal relationships and work hours domains were considered satisfactory. Costa et al.^28^ investigated 258 administration personnel at the Federal University of Acre, finding very satisfactory results for the interpersonal relationships domain and satisfactory results for the work hours domain. Pinto et al.^29^ assessed the QWL of 50 administrative personnel from a state higher education institution in Paraná, Brazil, finding very satisfactory results for the interpersonal relationships domain. In a study of 254 administrative personnel at the Federal University of Rio Grande do Sul, Mansano et al.^30^ found that the interpersonal relationships domain was considered satisfactory and the work hours domain was very satisfactory.

The interpersonal relationships domain of QWL is linked to the interaction process between workers and their superiors, colleagues, and subordinates; the way these interactions occur influences work performance.^19^ Considering that people’s social ties are affected by their income, Zhang & Xiang^31^ found that people with lower incomes tend to spend more time and engage more frequently in socializing than those with higher incomes. Thus, the results of the present sample indicate a healthy and collaborative work environment.

With face-to-face activities interrupted, work routines had to be adapted, and safety and protection initiatives against COVID-19 were announced to offer greater safety for everyone.^2,3^ However, the infection risk did not cease, and there was a high prevalence of COVID-19 infection in our sample. According to the National Household Survey, during the pandemic (2020), 26.8 million remote workers reported at least 1 symptom associated with COVID-19 infeciton.^6^

The pandemic affected perceived QoL, predisposing workers to demotivation, negativity, and exhaustion, which can also directly affect QoL.^13^ Thus, because of the pandemic’s effects on daily activities, the majority of participants considered their QoL and QWL worse than in the pre-pandemic period. Consonant with this finding, university professors in Rio de Janeiro reported worse health conditions in 2020 than 2019.^25^

These results demonstrate that the pandemic negatively affected QoL, and many negative effects on remote workers involve domestic, family, and work demands. Although working from home allows flexible work hours and less time spent commuting, it also involves serious problems with extended work days, more days worked, and an intensified work pace. Therefore, working from home does not necessarily guarantee greater job satisfaction.^8^

Our study has several limitations. First, as in all studies involving questionnaires, the accuracy of the results depends on the respondents’ honesty and memory. Second, sleep quality was not assessed, which may influence QoL. Third, the small sample size (n = 18) does not allow extrapolation to other populations. However, we believe that these limitations do not prevent us from drawing conclusions from the results. Despite being an innovative study during the COVID-19 pandemic, it was not intended to be exhaustive. On the contrary, proposals are needed to improve QWL among administrative personnel of public higher education institutions, especially remote workers, considering the short-, midand long-term impact of the pandemic.

## CONCLUSIONS

Income influences physical, emotional, and mental health, as well as interpersonal relationships and work hours, thus impacting the QoL of administrative personnel who worked from home during the COVID-19 pandemic. These findings can contribute to the development, implementation, or remodeling of institutional policies, work management, and health programs, since such problems contribute to worker absenteeism.

Considering the importance of determining the needs and potential of administrative personnel, instruments investigating QoL and QWL are important for prevention and intervention actions.
